# The importance of design in nanoarchitectonics: multifractality in MACE silicon nanowires

**DOI:** 10.3762/bjnano.10.204

**Published:** 2019-10-31

**Authors:** Stefania Carapezzi, Anna Cavallini

**Affiliations:** 1 Department of Physics and Astronomy, University of Bologna, Viale Berti Pichat 6/2, 40127 Bologna, Italy; 2 DEI-ARCES, Viale del Risorgimento 2, 40125, Bologna, Italy

**Keywords:** atomic force microscopy (AFM), capillary force, metal-assisted chemical etching (MACE), multifractal analysis, nanoarchitectonics, nanowires, self-assembly

## Abstract

**Background:** Mechanisms of self-assembly/self-organization are fundamental for the emergence of nanoarchitectonic systems composed by elemental units, and it is important to build a theoretical framework for them. Additionally, because the enhanced functionalities of these systems are related to their spatial morphologies, it is necessary to quantify the self-organized design through suited statistical analysis tools.

**Results:** We have investigated the self-assembly bundling process of nanowires fabricated by metal-assisted chemical etching (MACE). First, we have applied theoretical models in order to obtain a quantitative estimation of the driving forces leading to self-assembly. Then, we have studied the surfaces of the nanoarchitectures by means of multifractal analysis. We have found that these systems are not simple monofractals, but that the more complex paradigm of multifractality (different fractal dimensions across different scales) has to be applied to describe their morphology.

**Conclusion:** The multifractal analysis approach has proven its ability to discriminate among different MACE nanoarchitectures. Additionally, it has demonstrated its capacity to measure the degree of homogeneity of these surfaces. Finally, a correlation between the growth conditions and the capacity dimension of the nanowires was obtained.

## Introduction

In the last years, huge progress was made regarding the study and the technological exploitation of materials endowed with new properties deriving from their nanoscale features. In this respect, the field of nanoarchitectonics [1–2] has attracted attention as one of the most promising paradigmatic changes in nanotechnology. In general, the concept of nanoarchitectonics consists in the approach of building up large structures from nanoscaled units by self-assembly. This self-building is driven by the reciprocal interactions among the units, where these interactions are such as van der Waals, electrostatic, magnetic, molecular, and entropic forces [3]. The technological advantage is that in comparison to the nanoscaled units these self-organized assemblies possess new functionalities. Atoms, molecules, or even nanoparticles or nanowires (NWs) can be used as basic units to self-arrange in new wholes.

**Figure 1:**
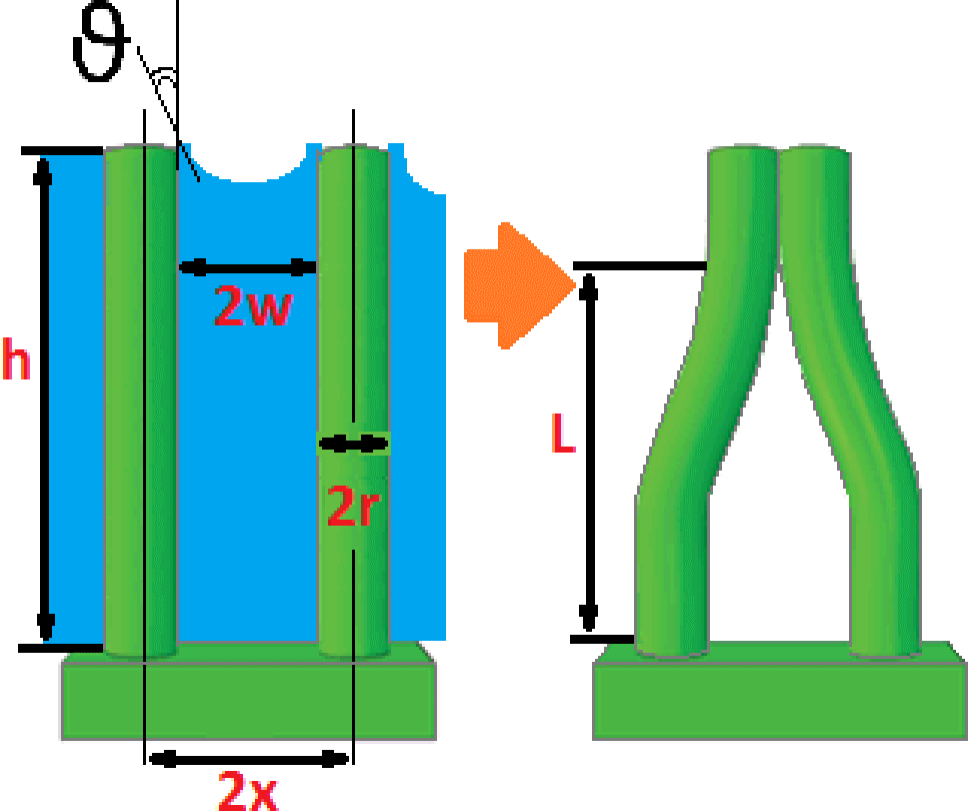
Schematic illustration of elastocapillary self-assembly of NWs induced by the surface tension between the residual fluid film and the NWs.

NWs are among the most widely investigated nanoscaled objects. Especially semiconductor NWs offer the unique promise to boost the performance of semiconductor devices by quantum effects. In this respect, silicon NWs [4–7] are key elements in the field of nanotechnology, given that they can be integrated in the microelectronic industry, which is mainly Si-based. From a technological point of view, it is essential to explore the possibilities of a large-scale fabrication of NWs. The top-down approach [8] represents the main route to achieve this goal, because it allows for wafer-scale growth by an easy adaptation of microfabrication equipment already available in the industry. The top-down methods involve the use of both dry [9–10] and wet etching [11] to carve nanostructures from a substrate. Metal-assisted chemical etching (MACE) [12–15] has gained particular attention in this regard, because it is simple, of low cost and versatile. MACE is an anisotropic wet etching technique where the sculpting of the nanostructures is catalyzed by a discontinuous thin film of noble metal deposited on a substrate. The metal works as a local cathode where the reduction of oxidants occurs. The underneath semiconductor is the local anode where a charge-mediated nucleophilic substitution reaction takes place, which causes silicon atoms to be etched/removed from the substrate. The metal layer, which is not consumed during the process, simply sinks down while the uncovered parts of the substrate form the tips of the NWs. Indeed, no consummation occurs when gold is used, while other metals are partly dissolved in many instances. Because the fabrication step occurs in liquid ambient a final drying step is inherently involved. Under certain conditions of 1) high NW density and 2) high aspect-ratio of NWs, the surface tension between the residual fluid film and the NWs could induce a self-assembly [16–17] (see Figure 1). The process of the assembly of NWs induced by elastocapillary forces is complex. There are many factors that influence the assembly such as periodicity, height, cross section, and tensile strength of the NWs as well as evaporation rate and the surface tension of the fluid. Elastocapillary self-assembly of NWs is an extensively investigated versatile and scalable method to design complex and robust surface nanoarchitectures [18]. For tuning and selectivity of the design of NW assemblies other approaches should be considered [19].

In hierarchical nanoarchitectures generated by NWs or other elemental nanobjects the self-assembly/self-organization mechanisms are pivotal to generate the assembled structures. In fact, the direct fabrication of such structures by microfabrication or even nanofabrication approaches would be challenging or impossible, given the nanosized dimensions of the basic units. Because the spatial layout is self-driven in contrast to a hetero-directed placement, asymmetric interaction potentials and entropic forces can lead to different aggregation schemes from place to place and across the scales of the generated structures. The control over the spatial arrangement of the assembled elements is a key issue in nanoarchitectonics, because the emerging functionalities of the whole are linked to its geometry. As a fundamental step towards the full command of the nanoarchitectonic design it is thus necessary to characterize the self-generated morphologies, in order to be able to discriminate among them and possibly to relate them to growth procedures from one side and to physical properties from another side. In this regard, fractal analysis [20] is an analytical framework fit for the purpose. Indeed, self-assembled patterns derived from aggregative processes, which are omnipresent in nature, have been characterized by their fractal dimension [21–23] that contains information about their geometrical structure at multiple scales. However, sometimes the richness of the organization of shape is such that it is impossible to describe it by just one scaling law. In this latter case a shift to multifractal analysis is necessary.

In the present work we show the results of multifractal analysis of nanoarchitectured surfaces of MACE Si NWs. The spontaneous arrangements of the NWs were investigated by using atomic force microscopy (AFM). Among the scanning probe techniques, AFM shows a peculiar capability to quantitatively characterize features with nanoscaled dimensions. To gain insight over the emergence of the organized nanoarchitectures we applied multifractal analysis to the AFM images. We have found that a single fractal dimension is not sufficient to describe the complex geometries of the NW systems. By examining the results of the multifractal analysis we have been able to highlight differences between the generated spatial patterns that we have correlated to the different growth conditions.

## A Brief Survey of Multifractal Analysis

Let the fractal object *F* be a subset of the *d*-dimensional Euclidean space 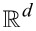, which is the physical support of *F*, and be covered by a *d*-dimensional grid of length scale ε. The box-counting (BC) fractal dimension, or capacity, *D*_BC_ is defined as

[1]
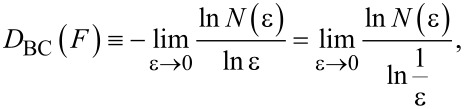


where *N*(ε) is the number of grid elements that overlap with *F*. Usually, *D*_BC_ is determined through the least squares linear fit of ln *N*(ε) as a function of ε. It is noteworthy to observe that Equation 1 represents a scaling rule that compares how much the detail (quantified by *N*) in a given pattern changes with the scale (ε), and it shows that for a (mono)fractal a single fractal dimension is able to characterize it across all the length scales [20]. For the deterministic fractals, which are mathematically constructed objects, the scale invariance holds for all scales. Well-known examples are Cantor set and Koch’s curve [20]. Instead, natural objects and phenomena are intrinsically finite and their fractality, if any, can be determined only within a specific regime of length scales. These structures are called random fractals.

When the fractal analysis is applied to investigate shapes of natural objects, this is performed by analyzing their images. Thus, it is necessary to reframe the above concepts within the field of image analysis. The simplest type of digital image is a binary image, that is a squared (for the sake of simplicity) *S* × *S* discrete matrix *M* of pixels where each pixel can have black or white colour. In this case the fractal object will correspond to the set of the pixels of a given colour, for instance black, while all pixels corresponding to the other colour will be disregarded. To evaluate the dimension of *D*_BC_, first a series of grids with different length scales will be overlaid to the image. Each grid is composed of *s*_ε_ × *s*_ε_ not overlapping boxes *G*_ε_(*i*,*j*), the size of which is the grid length scale ε, such that 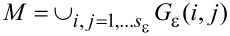. Then it is useful to introduce a local measure μ_ε_ (*i*,*j*), which amounts to the number of pixels belonging to the fractal object and contained in the box *G*_ε_(*i*,*j*). It is evident that, when a single global exponent characterizes a fractal object, the measure is uniform and 
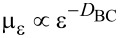
. However, in case of multifractal objects the above does not apply anymore and the measure μ_ε_ varies at different locations.

The quantitative description of multifractality can be performed in differrent manners. One approach passes through the calculation of the Lipshitz-Hölder exponent α, which gives account of the pointwise singularity of the object, and its distribution *f*(α), known as the multifractal spectrum. One method to determine *f*(α) is the following [24]. The probability distribution of μ_ε_ is introduced as

[2]
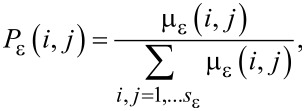


from which a one-parameter family of normalized measures is constructed:

[3]
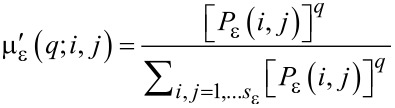


The parameter *q* works like a magnifying glass, enhancing 1) the regions of the fractal object with the lowest values of *P*_ε_(*i*,*j*) for *q <* 1 and 2) the regions with the highest values of *P*_ε_(*i*,*j*) for *q >* 1. The fractal dimension of the support of μ(*q*) is

[4]
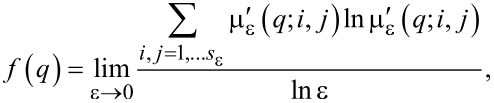


and the average value of the singularity strength


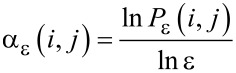


with respect to μ(*q*) is

[5]
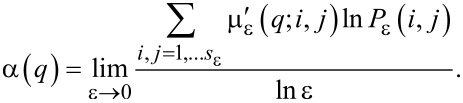


The mass exponent τ(*q*) is defined as

[6]
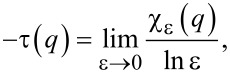


given that


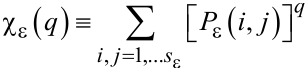


is the *q*-th power moment sum. τ(*q*) is linked to the multifractal spectrum by the Legendre transformation

[7]
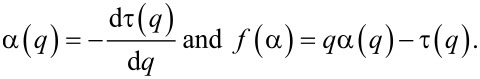


It is also connected to the generalized fractal dimensions [25–27]

[8]
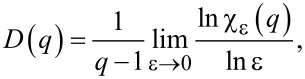


because τ(*q*) = (*q* − 1)*D*(*q*). Thus, an alternative way to determine the multifractal spectrum is to calculate *D(q)* from the above equation and to substitute it in Equation 7.

## Results and Discussion

### Elastocapillary self-assembly in MACE Si NWs

**Figure 2:**
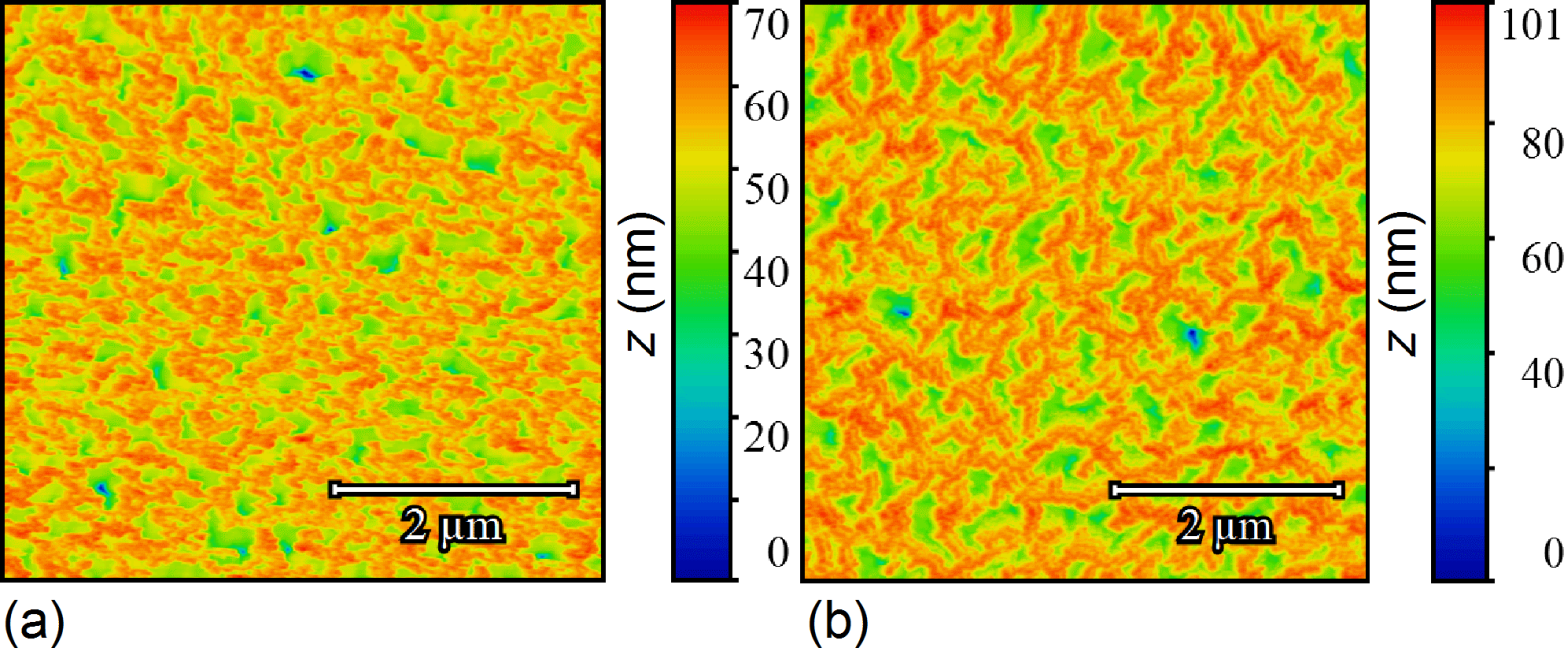
Representative AFM images of sample SiNW1 (a) and sample SiNW2 (b). The probed areas have a size of 5 × 5 μm^2^. The AFM measurements have been performed by using a NT-MDT Solver Pro 4H microscope, using tapping mode in ambient atmosphere and at room temperature.

Figure 2 shows typical AFM images of the MACE Si NWs investigated in the present work. The procedure to grow the MACE samples is carried out as follows: (100)-oriented Si wafers are the substrates. As a first step, the native oxide is removed from their surfaces by UV-oxidizing (2 min) and then dipping them (5 min) in 5% HF. Subsequently, 2 nm thick Au layers are deposited on the cleaned surfaces by electron beam evaporation. These gold films do not coat the substrates uniformly. The uncovered parts of the Si surfaces become the seeds of the NWs in the subsequent etching step. For the etching step, the substrates are immersed in an aqueous solution of HF (5 M) and H_2_O_2_ (0.44 M). Two types of MACE Si NWs have been synthesised, with differences in 1) the doping of the source substrates and 2) the etching time. Longer etching times yield longer NWs. For one sample (labelled from now on SiNW1; Figure 2a) the source substrate was P-doped with a doping density of 10^15^ cm^−3^ and a NW length of 5 μm. For the other sample (labelled SiNW2; Figure 2b) the source substrate was As-doped with a doping density of about 10^18^ cm^−3^ and a NW length of 1.3 μm.

**Figure 3:**
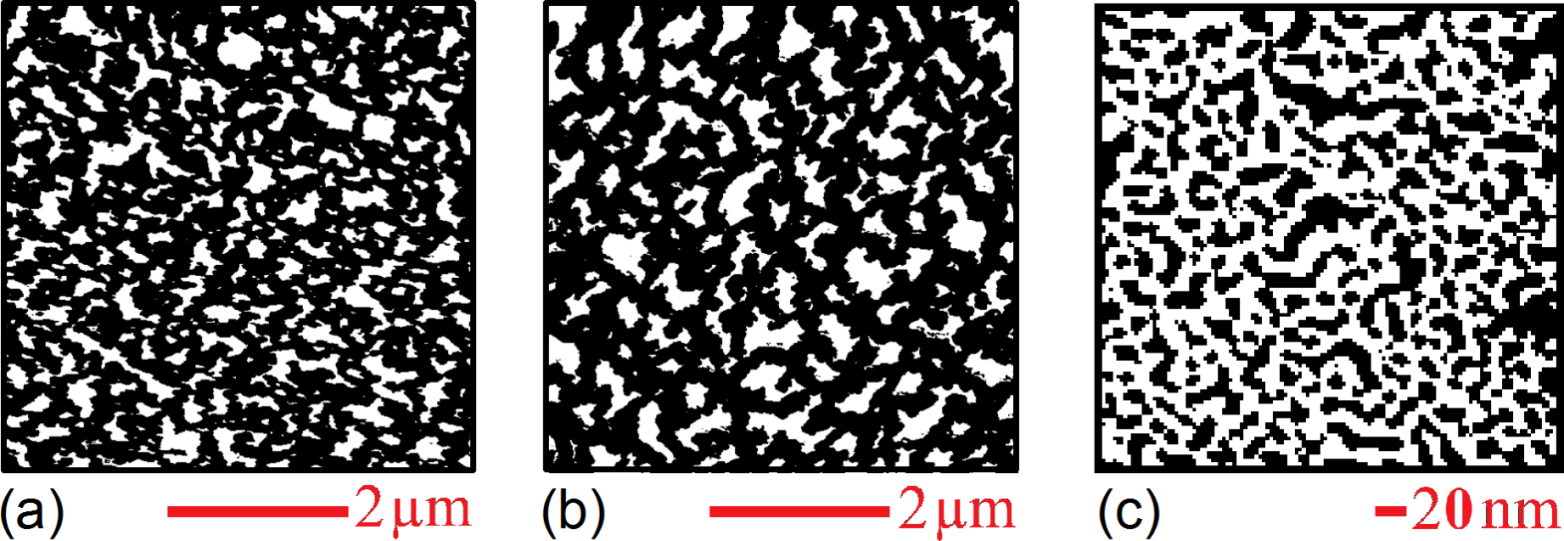
(a, b) Masks obtained by setting a threshold for the heights in the AFM images of SiNW1 and SiNW2 in Figure 2, in such a way that the black regions correspond to the tips of NWs. (c) Image obtained after binary-processing a SEM image adapted from [37] of a 2 nm Au layer deposited on a Si substrate by electron beam evaporation. The conditions during the growth of the gold thin film were similar to the ones during the growth of the samples SiNW1 and SiNW2. The black regions correspond to the uncovered silicon areas, while the white region represents the deposited Au. It is evident that by MACE processing the metal-coated substrate a spatially homogeneous arrangements of NWs would have been expected. On the contrary, in (a, b) the MACE-grown NWs are clustered.

Figure 3a and Figure 3b are the masked images obtained applying a height threshold on the AFM measurements of Figure 2. The threshold divides the surface into two regions: the tips of NWs (black colour) and the remainder of the sample (white colour). The NW tips appear clustered in both samples, creating a very complex architecture over the surfaces. It is noteworthy to observe that this clustering has not been purposefully induced by design of the locations of NWs, but it is a spontaneous assembly occurring during the NW growth. Recently, a direct observation of MACE NWs bending and sticking together during the drying step has been reported [28]. The authors have found experimentally that the bending/bundling of NWs depended on their aspect ratio. It occurred for aspect ratios greater than 1:10, while it was not observed for an aspect ratio of about 1:5. Actually, many more factors have impact on the self-assembly of arrays of nano- and microstructures with high aspect ratios, when a liquid is evaporated off the surface [29–30]. Considering two adjacent NWs, first 1) the capillary force between them should be able to overcome the elastic force moving them back to the original straight position, in order to bring them into contact during the evaporation of the liquid. Then a stable bundle will occur if 2) the adhesion force between the surfaces of the two NWs is larger than the elastic force. In order to theoretically confirm the self-assembly that occurred in both samples SiNW1 and SiNW2, we availed of literature [29,31] to model steps 1) and 2). Given a pillar-like structure of height *h*, clamped at one end, such that the other end is deflected by a length *w*, the magnitude of the elastic force acting on it can be calculated from the Euler–Bernoulli elementary beam theory [32], and it is given by

[9]
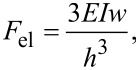


where *E* is the Young modulus of the pillar and *I* is its area moment of inertia. The magnitude of the capillary force *F*_C_ between two cylindrical pillars when partially immersed in a liquid is [29]

[10]
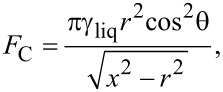


where γ_liq_ is the liquid surface tension, θ is the contact angle between the liquid and the surface of the pillar, *r* is the radius of the pillar and 2*x* is the interdistance between the axes of the two pillars (see Figure 1). We have availed of the above Equations 9 and 10 to estimate the magnitudes of the elastic and capillary forces in our case. To this aim we have determined average values of 4.5 nm and of 7.5 nm for *r* and *x*, respectively. Considering the data reported in literature, we have considered not a single value but a range of values of a) *E* = 80–120 GPa [33] and of b) γ_HF_ from 0.5 mN/m [34] to 10.2 mN/m [35]. A value of θ = 70° has been found for silicon surfaces and HF [36]. The range of values for the capillary force has thus been estimated to be 3–65 pN, which is orders of magnitude greater than the possible values of the elastic force, ranging from 0.9 to 1.4 fN for SiNW1, and from 53 to 80 fN for SiNW2. This confirms that under the growth conditions described here, *F*_C_ is greater than *F*_el_ and can bring the NWs into contact.

In order to find out whether the formed NW bundles are stable, we have calculated the critical aspect ratio [31] of the NWs of samples SiNW1 and SiNW2. The critical aspect ratio is a threshold value for the stability of the bundles. NWs with aspect ratios larger than the critical one will remain attached after bending towards each other and getting in contact. Given two collapsed pillars, such that the length of the non-contact portion is *L* (Figure 1b), a critical value of *L* can be estimated from [31]

[11]
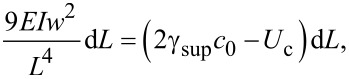


where the term on the left-hand side is the decrease in strain energy if the non-contact region is increased by d*L*, and the term on the right-hand side is the energy required to separate the surfaces of the two pillars by d*L*. γ_sup_ is the surface energy of the pillar, *c*_0_ is the contact width at equilibrium of the two pillars under no external force, and *U*_c_ is the stored elastic energy normalized to the contact length due to the deformation near the contact region. The values of *c*_0_ and *U*_c_ are given by [31]

[12]
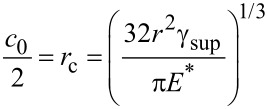


and

[13]
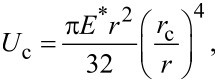


given that E* = *E*/(1 − ν^2^) where ν is the Poisson’s ratio. By rearranging Equation 11 it is possible to derive the critical aspect ratio as

[14]
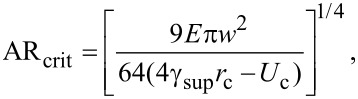


since *I* = π*r*^4^/4 for a cylindrical pillar. Again, a range of values for γ_sup_ from 1 to 2.2 J/m^2^ has been used according to literature [38], and a Poisson’s ratio of 0.22. The values of *AR*_crit_ thus obtained range from 2.2 to 1.5, while the aspect ratios of the NWs are of 555 for sample SiNW1 and of 144 for sample SiNW2. This confirms theoretically the stability of the observed NW clusters. The process of self-assembly yielded the hierarchically structured MACE NW surfaces as shown in Figure 2. The complex spatial design thus achieved has been investigated by means of multifractal analysis.

### Multifractal analysis applied to MACE NWs

**Figure 4:**
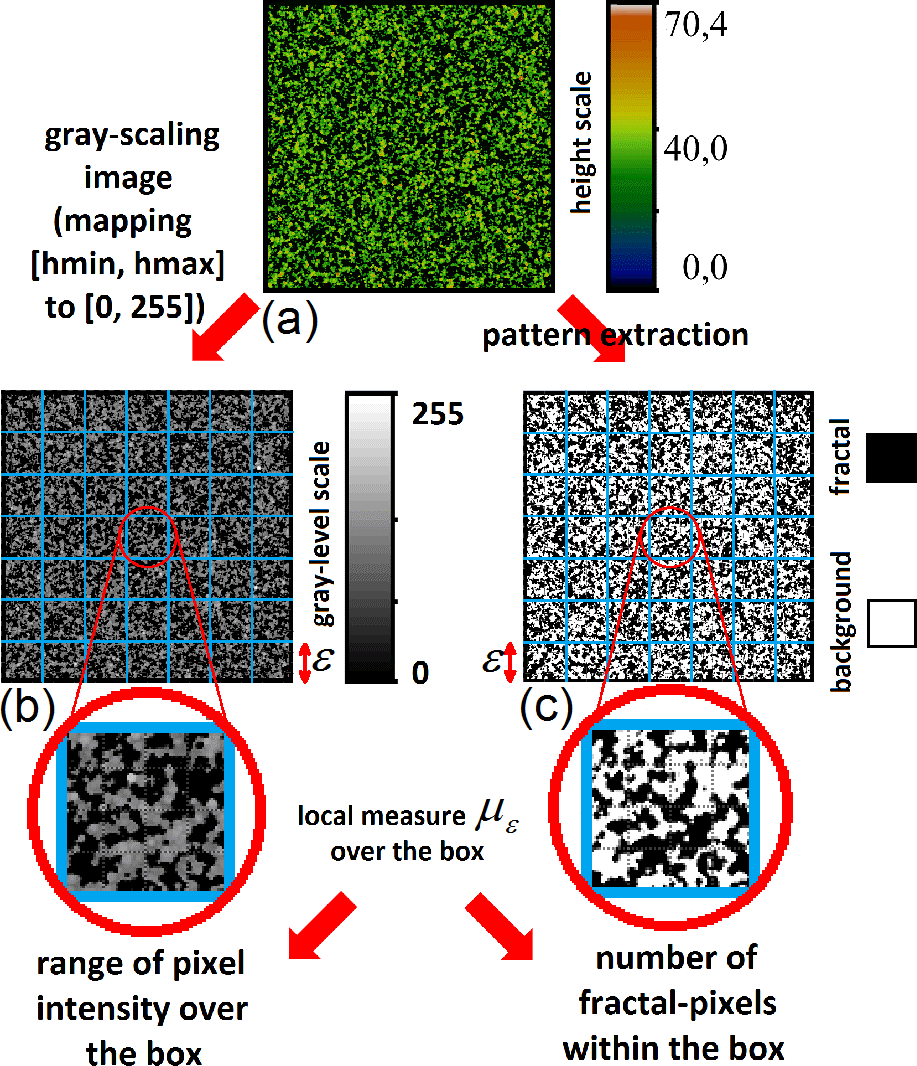
(a, b) Schematic of the differential box counting method applied to the AFM measurements. Each AFM image (a) is converted into a grey-scaled image (b). For a given grid of unit length ε laid down over the image (blue lattice), the local measure μ_ε_(*i*,*j*) is computed as the range in intensity of all pixels belonging to the box. For the sake of comparison, the flow chart for the more common box counting method over binary images is illustrated in panels (a) and (c). From the AFM image (a) is extracted a silhouette or a mask (c). The region subject to fractal analysis is black (in the present case), while all remaining pixels are set to white. For a chosen grid, the local measure μ_ε_(*i*,*j*) is then computed as the sum of black pixels within the box.

In a previous section we gave a brief survey of the computational procedures involved into performing fractal and multifractal analysis. In both kinds of analysis, after the preliminary step of overlaying a grid of length scale ε to the image, it is crucial to establish a rule to assign a value to the local measure μ_ε_(*i*,*j*) over each box of the grid. However, the recipe that has been given, μ_ε_(*i*,*j*) ↔ number of pixels belonging to the fractal object and contained in the box (*i*,*j*) is meaningful only for binary images. Indeed, fractal objects that can be described by binary images are, for example, 2D contours or the 2D correspondent filled patterns (such as the regions of homogeneous colour of Figure 3). These fractals are clearly just a subset of the possible random fractal phenomena or structures that can be met in nature, where the complex features of real 3D morphologies cannot be rendered by binary images. In fact, the AFM images of Figure 2 are RGB images, where a colour scale connects the image colours to height values. It is noteworthy to observe that, once a suitable mapping is established to compute the local measure μ_ε_ for RGB or grey-scale images, the steps already described to calculate the fractal dimension, the multifractal spectrum or the generalized dimensions would be the same. In the present work we have chosen to use grey-scaled versions of the AFM images to perform the multifractal analysis, which has been implemented by means of the FracLac plugin [39] of the image analysis software ImageJ [40]. In fact, in the grey-scaled AFM images the interval of heights actually measured is mapped onto grey-level values ranging from 0 (black) to 255 (white), which corresponds to a simple rescaling. The flow chart of the followed procedure has been schematically illustrated in Figure 4a,b. To calculate the local measure μ_ε_ for the grey-level images we availed of the differential box counting method: μ_ε_(*i*,*j*) = Δ_ε_*I*(*i*,*j*) + 1, given that Δ_ε_*I*(*i*,*j*) = *I*_max_ − *I*_min_ is the difference in pixel intensities (grey-level values) over the box (*i*,*j*).

**Figure 5:**
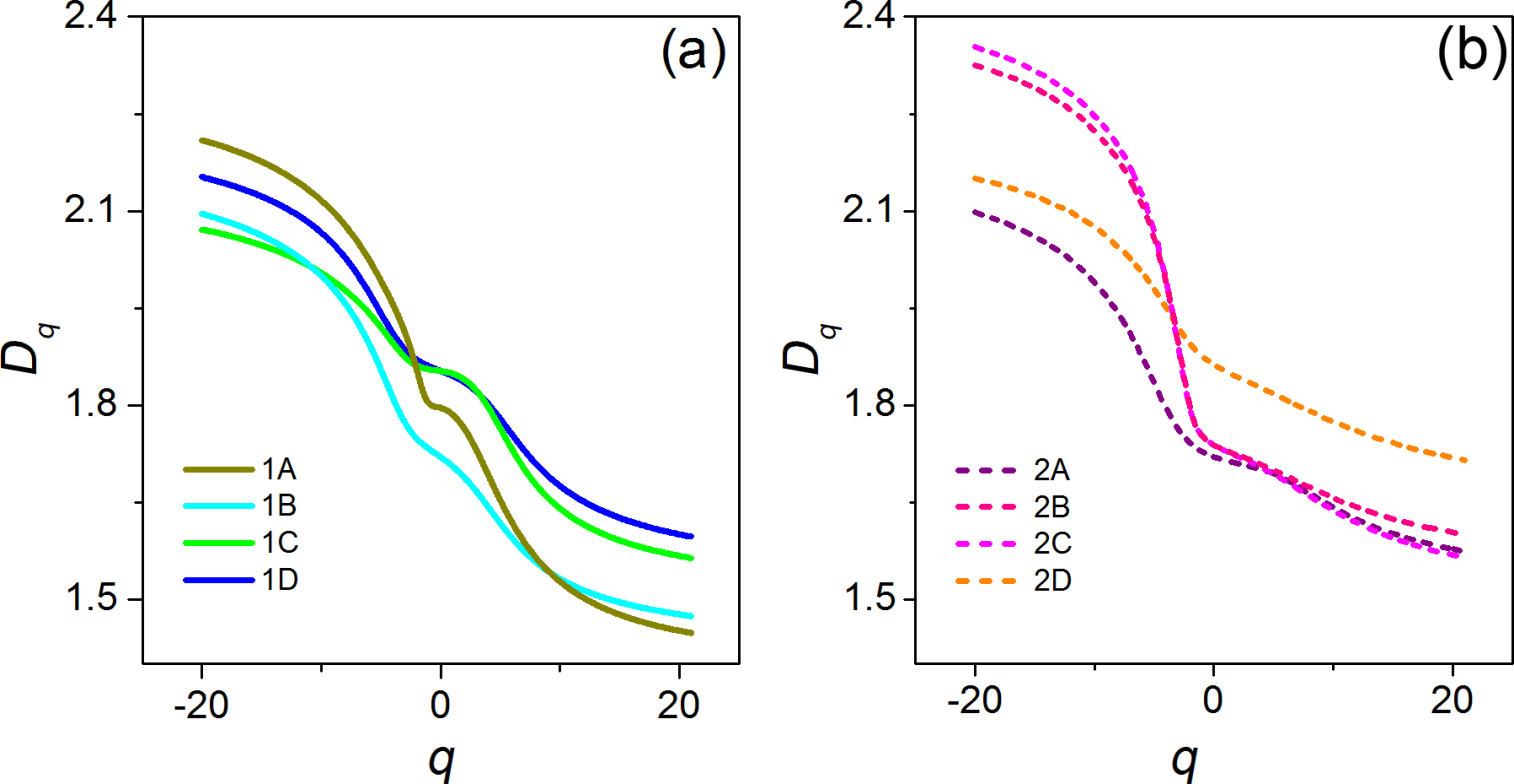
Curves of the generalized dimension *D_q_* as a function of *q* for different probed areas of samples SiNW1 (a) and SiNW2 (b). The values of *D*_0_, *D*_1_ and *D*_2_ for these areas have been reported in Table 1.

Figure 5 shows the *D_q_* curves of different sampled areas of the surfaces of samples SiNW1 (a) and SiNW2 (b). First of all, these graphical spectra of *D_q_* show that the nanoarchitectured surfaces generated by the self-assembly of NWs are indeed multifractal. Monofractals or objects that are not fractals tend to have flatter *D_q_* curves than multifractals. Ideally, a *D_q_* curve is flat for a monofractal because 
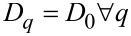
 [25]. With reference to Equation 8, it should be remembered that the generalized dimension is linked to the probability distribution *P*_ε_(*i*,*j*). The largeness of an element of such a distribution is directly related to the correspondent largeness of μ_ε_(*i*,*j*) (see Equation 2). Thus, the parameter *q* is a kind of a resolution parameter that enhances 1) regions corresponding to higher μ_ε_ values for positive values of *q*, and 2) regions of lower μ_ε_ values for negative values of *q*. Keeping this in mind, and taking into account how we have defined above the local measure μ_ε_, we observe that for sample SiNW1 the *D_q_* values for each *q* are lower than the ones for sample SiNW2, or they are in the same range. That is, in sample SiNW2 there is a tendency to higher fractality for both areas with larger or smaller ranges of pixel intensities Δ_ε_*I*. This is related to the different growth conditions of sample SiNW1 and sample SiNW2.

In Table 1 are reported the *D*_0_, *D*_1_ and *D*_2_ values of the sampled areas from sample SiNW1 and SiNW2. It can be proved that 
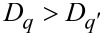
 if *q*’ *> q*[25], and in fact we have found that *D*_0_ *> D*_1_ *> D*_2_, where the difference between these values is an indication of the multifractality of the surfaces of samples SiNW1 and SiNW2. *D*_0_ is the so called capacity (or box counting) dimension that would coincide with *D*_BC_ in a monofractal system. In our case we have found values of *D*_0_ of about 1.84 for most of sampled areas of sample SiNW1, while a value of 1.72 was obtained in one case. In contrast, for sample SiNW2 we obtained values of about 1.73, again with a value of 1.86 obtained in one case. Such differences in capacity dimension for different areas of the same sample may be indicative of a certain degree of inhomogeneity in the final design of the NW patterns. Nevertheless, it is interesting to observe that the range of possible *D*_0_ values seems to be approximately the same in both samples. It has to be noted that MACE Si NW samples have been characterized by simple fractal analysis in a previous work [37], where a value of *D*_BC_ of about 1.9 has been found. However, it should be taken into account that in that case the analysis has been performed over binarized SEM images, where the patterns under study have been the regions corresponding to the tops of NWs, similar to the masked regions of Figure 3b,c. Thus, the complex 3D arrangements of the NWs have been completely ignored.

**Table 1:** Main parameters obtained from the multifractal analysis of the sampled areas of SiNW1 and SiNW2.

SiNW1			

area	*D*_0_	*D*_1_	*D*_2_
1A	1.7958	1.7854	1.7629
1B	1.7197	1.7054	1.6878
1C	1.8528	1.8486	1.8394
1D	1.8528	1.8453	1.8352
SiNW2			

area	*D*_0_	*D*_1_	*D*_2_
2A	1.7197	1.714	1.71
2B	1.8628	1.8526	1.8435
2C	1.7378	1.7289	1.7209
2D	1.7378	1.7296	1.7225

**Figure 6:**
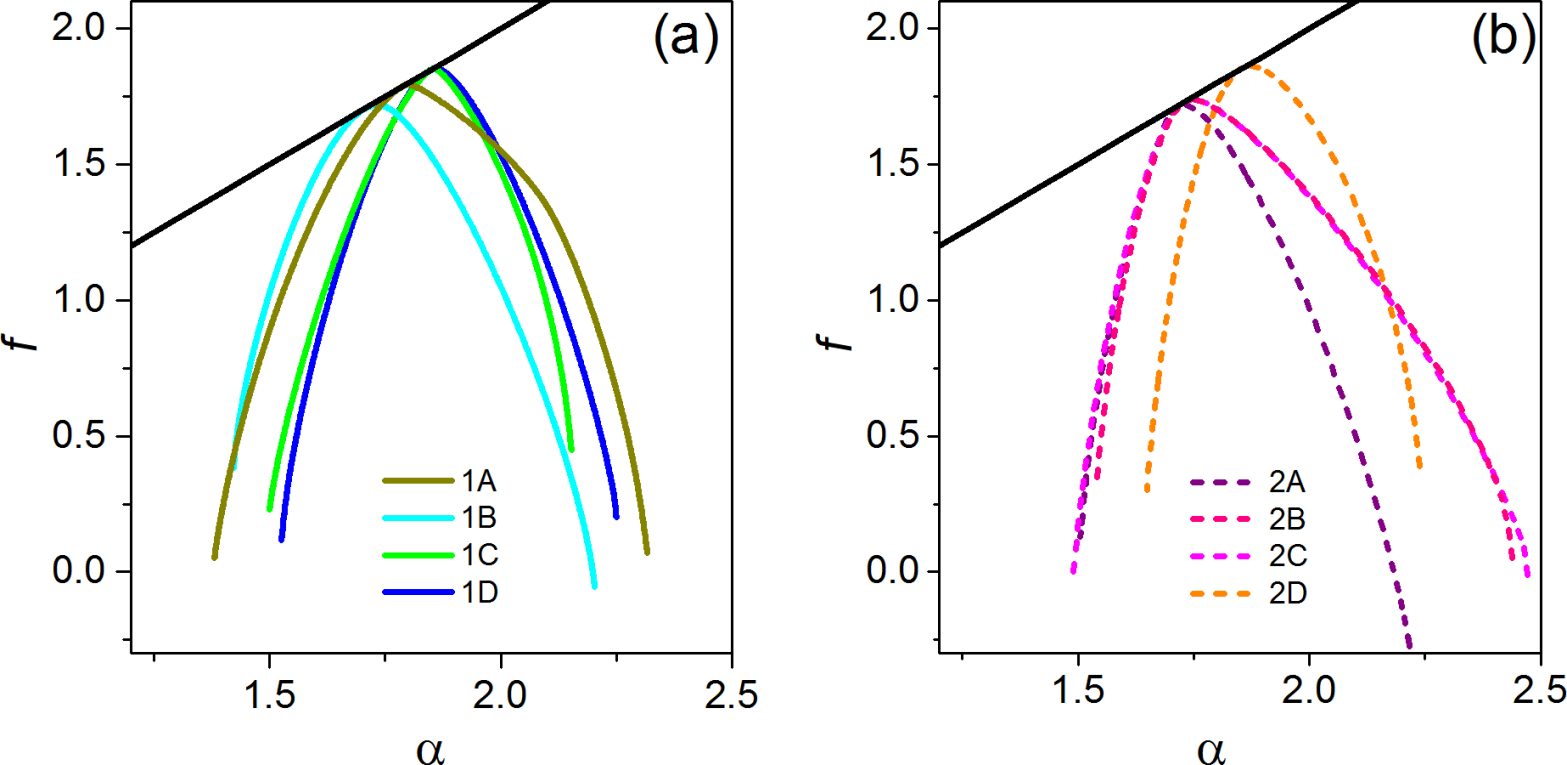
Multifractal spectra of sampled areas of MACE SiNW1 (a) and SiNW2 (b). The black line is the first diagonal.

Finally, in Figure 6 the multifractal spectra *f*(α) of all the sampled areas of samples SiNW1 (a) and SiNW2 (b) are reported. Again, there is an indication of multifractality in both samples, given that in the case of an ideal monofractal *f*(α) would reduce to just a single point. In contrast to *D_q_*, which represents the various dimensions of the distribution of the ranges of pixel intensity values over the whole imaged area, *f*(α) is the dimension obtained over different sub-regions that display the same α. Each curve *f*(α) shows the characteristic convex shape, peaked at α(0), where *f*(α(0)) = *D*_0_, as can be easily verified by Equations 6–8. The α values to the left of α(0) are associated with positive *q* values, the ones to the right of α(0) are associated with negative *q* values. Thus, the presence or the absence of symmetry of *f*(α) around its peak mirrors the same kind of symmetry/asymmetry in the distribution between regions with a large/small range of pixel intensity values. From Figure 6 it appears that *f*(α) spectra of the sampled areas of SiNW1 have a tendency to be symmetric. Instead of this, in the case of sample SiNW2 the intervals of α values of sub-areas with a smaller range of pixel intensity values (right part of the curve) are bigger than the ones for sub-areas with a larger range of pixel intensity values (left part of the curve). It can be suggested that for sample SiNW2 the multifractality is enhanced in sub-areas with smaller range of pixel intensity, that is of smaller height variation. In fact, it should be remembered that the pixel intensity is simply a rescaling of the measured height value. It is interesting to note that the property of symmetry/asymmetry of the *f*(α) spectrum applies to all areas of each sample, appearing to be an intrinsic feature.

## Conclusion

In this work we have investigated the spontaneous spatial organization of nanoarchitectures of MACE Si NWs. First, we have applied theoretical models in order to estimate the driving forces leading to the self-assembly. Then we have availed of multifractal analysis to analyze the patterns. Our results confirm that fractal analysis would not be sufficient to capture the whole richness of the self-assembled structures. Differences in growth conditions result in differences in the generalized dimension and the multifractal spectrum. In contrast, when the same quantities are calculated over areas of the same sample the results show coherence, even if a minority of cases departs. This means that the nanoarchitectonic surfaces present locally some degree of inhomogeneity. This also highlights multifractal analysis as a powerful tool to “measure” the design of fractal-like nanoarchitectures. Finally, a correlation has been found between the growth conditions and the tendency to multifractality, which is more uniform across the MACE nanoarchitectures of sample SiNW1, while it is accentuated in the region formed by the tips of the nanowires of sample SiNW2.
